# The Effects of Dietary Manganese and Selenium on Growth and the Fecal Microbiota of Nursery Piglets

**DOI:** 10.3390/vetsci10110650

**Published:** 2023-11-10

**Authors:** Clint E. Edmunds, Christina B. Welch, Jeferson M. Lourenco, Todd R. Callaway, T. Dean Pringle, C. Robert Dove

**Affiliations:** 1 School of Sciences, Clayton State University, Morrow, GA 30260, USA; 2 Department of Animal and Dairy Science, University of Georgia, Athens, GA 30602, USA; christina.welch@uga.edu (C.B.W.); jefao@uga.edu (J.M.L.); todd.callaway@uga.edu (T.R.C.); crdove@uga.edu (C.R.D.); 3 North Florida Research and Education Center, Institute of Food and Agricultural Sciences, University of Florida, Quincy, FL 32351, USA; td.pringle@ufl.edu

**Keywords:** growth performance, manganese, microbiome, nursery, selenium, swine, *Streptococcus*, *Roseburia*, *Turicibacter*

## Abstract

**Simple Summary:**

Piglets experience great stress when they are weaned from their mothers at about three weeks of age. One of those types of stress occurs at a cellular level and can impact what types of bacteria can grow and live in the piglets’ digestive systems. There are certain enzymes that fight cellular stress, and certain ingredients (manganese and selenium, two mineral feed ingredients) can be provided to the weaned piglets to boost their stress defense. The objective of this study was to demonstrate how increased mineral concentration in the diet can impact how the pig grows and how it affects the piglets’ digestive systems. This study provides preliminary evidence that a specific mineral, manganese, can impact piglet growth and the gut of the pig in a positive way by decreasing bacteria that are considered to be pathogenic or “bad” and increasing bacteria that are considered to be beneficial or “good”. In this study, selenium had no significant impacts on animal or bacterial growth. This study can be used as a foundation for future research that can dive into greater detail into further application of these minerals in the field of animal nutrition.

**Abstract:**

The objective of this study was to determine the impact of varying dietary manganese and selenium concentrations, antioxidant cofactors, on the growth performance and fecal microbial populations of nursery pigs. The piglets (*N* = 120) were blocked by weight (5.22 ± 0.7 kg) and sex. The pens (*n* = 5/treatment) within a block were randomly assigned to diets in a 2 × 3 factorial design to examine the effects of Se (0.1 and 0.3 mg/kg added Se) and Mn (0, 12, and 24 mg/kg added Mn) and were fed in three phases (P1 = d 1–7, P2 = d 8–21, P3 = d 22–35). The pigs and orts were weighed weekly. Fecal samples were collected d 0 and 35 for 16S rRNA bacterial gene sequencing and VFA analysis. The data were analyzed as factorial via GLM in SAS. There was a linear response (*p* < 0.05) in overall ADG across dietary Mn. Supplementing 24 mg/kg Mn tended to decrease (*p* < 0.10) the relative abundance of many bacteria possessing pathogenic traits relative to Mn controls. Meanwhile, increasing Mn concentration tended to foster the growth of bacteria correlated with gut health and improved growth (*p* < 0.10). The data from this study provide preliminary evidence on the positive effects of manganese on growth and gut health of nursery pigs.

## 1. Introduction

Piglets are exposed to a number of physiological stressors in response to nutritional, social, and immunological changes that occur as a result of being weaned from sows [1]. In addition, oxidative stress plays a large role in the physiological challenges that affect piglets at weaning [1,2] and is the result of reactive oxygen species (ROS) accumulating intracellularly and impairing ordinary cell function in response to stress [3,4,5,6,7]. The accumulation of ROS can result in neurological disorders, irregular cell growth, and several forms of cancer in both humans and pigs [2,5,8,9]. As a defense mechanism, specific enzymes are synthesized by the pig to fight these destructive ROS [10]. Three antioxidant enzymes are considered to be the most active: manganese-superoxide dismutase (MnSOD), glutathione peroxidase, and catalase [9]. MnSOD, a mitochondrial antioxidant, uses manganese (Mn) as a cofactor for catalyzing the dismutation or molecular reorganization of the superoxide anion into hydrogen peroxide or molecular oxygen products; these products are then metabolized or eliminated by the body [6,7,8,9,10,11]. Glutathione peroxidase utilizes the trace element selenium (Se) to convert the ROS hydrogen peroxide into water, which is subsequently eliminated [12,13]. Therefore, altering supplemental dietary concentrations of manganese and selenium could affect the antioxidant status of nursery pigs and, as a result, improve growth performance.

In swine, weaning is often linked with profound changes in the gastrointestinal tract microbiota and host immune activity [14]. Immediately following weaning, piglets experience a period of sub-optimal growth and an increased incidence of intestinal disturbances with diarrhea (e.g., post-weaning *E*. *coli* diarrhea) [14]. These challenges have caused producers to utilize antibiotics such as Carbodox [15,16], which may add to the world-wide dissemination of antimicrobial resistance. There are bans on the use of in-feed antibiotics among many countries, including those in the European Union [14]. This necessitates the need for finding antibiotic-like feed ingredients for swine diets. Limited research exists on the impact that supplementing dietary manganese and selenium to nursery pigs has on their gut microbial populations and/or gut health. Manganese and selenium are important dietary microminerals to include due to the physiological roles mentioned and previously reported [17,18,19,20,21,22]. Due to these important physiological roles, we hypothesized that dietary alterations of Mn and Se would modulate the microbial population of piglets, and as a result, may impact performance and the overall gut health of those young piglets. Thus, the objective of this study was to evaluate the impact of altering supplemental dietary concentrations of manganese and selenium in the basal diets of nursery pigs on growth characteristics and fecal microbiota profile.

## 2. Materials and Methods

### 2.1. Animal Care and Experimental Design

This study was conducted in conjunction with Edmunds et al. [23], which measured the impacts of manganese and selenium supplementation on growth performance and oxidative stress. All care, handling, and sampling procedures of nursery piglets in the present research study were approved by the Institutional Animal Care and Use Committee at the University of Georgia prior to the beginning of this nursery trial (AUP#: A2018 08-012-A1). The weaned piglets (*N* = 120; 5.22 ± 0.7 kg; 21 ± 3 d old) were blocked by weaning weight and balanced by sex (2 gilts and 2 barrows per nursery pen; 4 piglets per nursery pen). The pens (*n* = 5/dietary treatment) were in a climate-controlled nursery room at the University of Georgia in the Large Animal Research Unit, and the piglets consumed water and feed ad libitum for 35 d following weaning. Trace mineral premixes were formulated and mixed in a way that resulted in a 2 × 3 factorial arrangement of treatments: two concentrations of selenium (0.1 and 0.3 mg/kg) and three concentrations of manganese (0, 12, and 24 mg/kg). The trace mineral premixes had an inclusion rate of 1% of the basal nursery diets. The composition and analysis of the basal diets (Table 1) were previously described by Edmunds et al. [23]. The basal diets were formulated to meet the current NRC requirements for all other nutrients and trace minerals [24]. Pelleted basal dietary treatments were assigned randomly within each weight block on d 0. The dietary treatments were fed in three phases (Table 1): Phase I from d 0–7, Phase II from d 8–21, and Phase III from d 22–35. The body weights and feed intake of the nursery piglets were recorded weekly to ensure that the appropriate growth and feed intake were occurring, but overall (d 0–35) growth performance data were presented (average daily gain (ADG), average daily feed intake (ADFI), and feed efficiency (G:F)).

### 2.2. Fecal Collection and Storage

The fecal samples were obtained on d 0 and 35 of the study from individual piglets using a sterile cotton swab to stimulate fecal excretion. The samples were placed in 50 mL conical tubes and frozen at −20 °C until further DNA processing could be performed on the fecal samples. The pen was the experimental unit for growth performance data, therefore, the samples were pooled by pen prior to DNA extraction.

### 2.3. DNA Extraction and Sequencing

Deoxyribonucleic acid (DNA) was extracted from the fecal samples following the procedures described in detail by Welch et al. [25] with some minor modifications. Following extraction, the fecal samples were shipped to LC Sciences (Houston, TX, USA) for library preparation and 16S ribosomal ribonucleic acid (rRNA) gene sequencing, following the protocol described by Welch et al. [25].

### 2.4. Volatile Fatty Acid Analysis

The analysis of volatile fatty acid (VFA) concentrations was accomplished via the protocol established by Lourenco et al. [26] using gas chromatography.

### 2.5. Statistical Analyses

All growth performance analyses were carried out using the pen as the experimental unit and weight as the experimental blocking factor. The initial body weight was used as a covariate for all growth performance parameters that were analyzed. The performance data were analyzed in a 2 × 3 factorial design via PROC GLM in SAS 9.4 (SAS Enterprise, Cary, NC, USA). A linear orthogonal contrast was used for Mn concentration. The indices of alpha diversity (operational taxonomic units (OTUs), Shannon, evenness) and bacterial abundances at the phylum, genus, and species level were run as a 2 × 3 factorial design utilizing the same analysis program, experimental unit, and blocking factor. In addition, the day was included into the model to detect differences attributed to sampling time and any interaction of day with manganese and selenium. Statistical significance was established at *p* < 0.05 and statistical tendencies were considered at 0.05 < *p* < 0.10.

## 3. Results

### 3.1. Animal Performance

Selenium and the interaction between Mn and Se did not have an effect on any growth performance parameters measured (*p* > 0.14; Table 2). However, Mn supplementation increased average daily gain (*p* = 0.007) in a linear manner (*p* < 0.05). There were no differences observed between treatments in body weight observed on d 0 or 35, average daily feed intake, or the ratio of gain-to-feed (*p* > 0.10).

### 3.2. Volatile Fatty Acids and Alpha Diversity

There was no significant interaction or main effect on any volatile fatty acid parameter except the molar proportion of acetate (Table 3). There was a significant Mn × Se interaction and Mn × day interaction on the molar proportion of acetate (*p* < 0.05); however, the molar proportion of acetate did not differ on d 35. Mn and Se supplementation did not have an effect on any of the alpha diversity indices measured: OTUs, Shannon, and evenness indices (Table 3).

### 3.3. Microbial Populations

**Figure 1:**
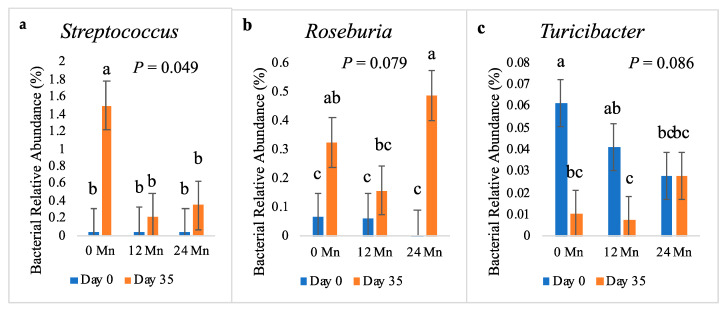
The effect of varying dietary manganese on bacterial relative abundance for the genera *Streptococcus* (**a**), *Roseburia* (**b**), and *Turicibacter* (**c**) in nursery pigs (*n* = 10 pens/Mn treatment). *p* values reported for Mn × Day effect. ^abc^ indicates significant differences (*p* < 0.05). Mn supplementation levels (mg/kg) are abbreviated as Mn 0, 12, and 24, respectively.

Because Mn affected average daily gain, the present study focused on how the microbial population changed over the post-weaning period based on Mn level (*n* = 10 pens/Mn treatment); however, information on the highlighted bacterial genera regarding Se supplementation can be found in the supplemental materials (Supplemental Figure S1). There was a higher relative abundance of the genus *Streptococcus* in the non-Mn supplemented piglets on d 35 compared to the piglets supplemented with Mn (*p* = 0.049; Figure 1) that did not occur in the feces of Mn supplemented piglets. Mn supplementation tended to impact the relative abundance of *Roseburia* (*p* = 0.079) on d 35 with the highest relative abundance being in the feces of the piglets who received the highest Mn supplementation (24 mg/kg). *Turicibacter* populations decreased in the non-supplemented and 12 mg/kg Mn piglets from d 0 to d 35; however, the piglets receiving the most Mn supplementation (24 mg/kg) did not have a decrease in *Turicibacter* from d 0 to d 35 (*p* = 0.086).

**Figure 2:**
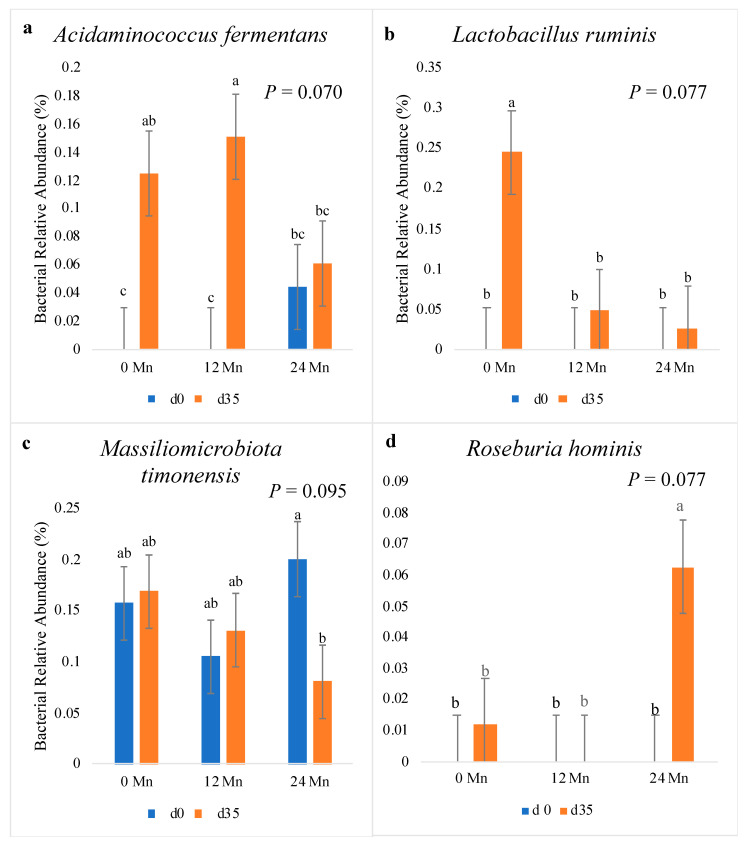
The effect of varying dietary manganese on bacterial relative abundance for the genera *Acidaminococcus fermentans* (**a**), *Lactobacillus ruminis* (**b**), *Massiliomicrobiota timonensis* (**c**), and *Roseburia hominis* (**d**) in nursery pigs (*n* = 10 pens/Mn treatment). *p* values reported for Mn × Day effect. ^abc^ indicates significant differences (*p* < 0.05). Mn supplementation levels (mg/kg) are abbreviated as Mn 0, 12, and 24, respectively.

In the present study, supplementary dietary Mn tended to affect the relative abundance of *Acidaminococcus fermentans* (*p* = 0.070; Figure 2) over time. The piglets receiving the most Mn supplementation (24 mg/kg) did not have a greater relative abundance of *A. fermentans* from d 0 to 35 (*p* > 0.05); whereas, the non-supplemented and lower supplementation (12 mg/kg) piglets had a relative abundance increase from d 0 to 35 (*p* < 0.05). Mn supplementation tended to have an effect on the relative abundance of *Lactobacillus ruminis* (*p* = 0.077) from d 0 to 35. On d 35, the relative abundance of *L. ruminis* was significantly increased (*p* < 0.05) in piglets fed 0 mg/kg Mn compared to those fed 12 mg/kg or 24 mg/kg Mn. The relative abundance of *Massiliomicrobiota timonensis* was impacted by Mn supplementation over time (*p* = 0.095). On d 35, the relative abundance of *M. timonensis* decreased in 24 mg/kg piglets compared to d 0 (*p* < 0.05); whereas there was no change in relative abundance in either 0 mg/kg and 12 mg/kg Mn piglets from d 0 to 35 (*p* > 0.05). The relative abundance of *Roseburia hominis* was affected by Mn supplementation over time (*p* = 0.077). The piglets receiving the highest Mn supplementation (24 mg/kg) had an increase in the relative abundance of *R. hominis* from d 0 to 35 in the feces (*p* < 0.05), whereas the other dietary groups did not.

## 4. Discussion

There was not a significant effect of selenium on nursery piglet growth performance parameters or the fecal microbiome. In this study, there was not a negative impact from the lower selenium concentration diets on growth performance due to the presence of adequate vitamin E in the basal diets.

While the supplementation of dietary manganese after the nursery stage of production has drawn research interest, minimal research with Mn has been carried out with piglets immediately following the weaning stage of production. The modern NRC recommendations for provision of Mn is 4 mg/kg supplemented for pigs that weigh 5–11 kg, 3 mg/kg supplemented Mn for pigs weighing 11–25 kg, and even less as the pigs grow even larger [24]. Inclusion levels above the published requirements have demonstrated positive impacts on the growth performance characteristics of pigs. Grummer et al. [27] reported that including 40 mg/kg Mn in grower pig diets improved ADG and G:F compared to control pigs fed no Mn. Edmunds et al. [23] reported improvements in ADG and oxidative status upon supplementation of nursery piglets above the nutritional requirement at 12 and 24 mg/kg Mn. By comparing the previously referenced study and the present study, there are some important data that can be connected, especially as they relate to how weaning pigs deal with ROS via manganese-superoxide dismutase and how that impacts piglet growth [23]. There have been effects on growth reported in sows and piglets as a result of dietary Mn supplementation [19,28]. It was reported that supplementing sows with 20 and 40 mg/kg Mn during gestation and lactation can have a positive impact on the ADG of piglets while in the farrowing room, as well as increase the weaning weight of piglets [29]. It was also determined that piglets had a more primed antioxidant response in specific tissues at weaning if the sow was supplemented above the NRC recommendation during the gestation and lactation periods [29]. The current study supports these previous findings that the inclusion of supplemental Mn above the NRC requirement may have significant implications for the swine industry by increasing piglet growth (ADG) during the nursery stage of production systems.

Although there were no direct effects of Mn on microbial diversity as a whole, Mn supplementation did impact specific bacterial relative abundances that could ultimately have a significant contributing role in the overall health of the piglets. *Streptococcus* is generally considered to be a pathogenic genus in most food animal species and humans. There are various *Streptococci* that are of ecological importance to the microbial flora of humans and animals; but there are also some that cause disease and can be harmful in humans and animals [30]. Torres-Pitarch et al. [31] found that ileal *Streptococcus* was negatively correlated to growth. Supplemental dietary copper and zinc decreased colonic digesta levels of *Streptococcus*, along with other pathogenic genera such as *Enterobacter* and *Escherichia* [32]. It is known that copper and zinc have growth promoting [33,34] and antimicrobial effects [35,36]. Less is known about manganese and its potential effects on the gut microbiome; however, this study suggests that Mn has a similar impact by increasing ADG while decreasing the fecal relative abundance of *Streptococcus*. This study may serve as preliminary evidence that supplemental dietary manganese may have some antimicrobial effects on the genus *Streptococcus*, especially following a five-week nursery period.

The genus *Roseburia* produces short-chain fatty acids from the metabolism of complex polysaccharides, especially butyrate, which can positively affect gut motility, maintain host immunity, and decrease inflammatory properties [37,38]. *Roseburia* is a common genus found in the gut of nursery piglets and is generally considered a beneficial genera for the host [39,40]. Though little research has directly studied the effects of dietary manganese on the presence of *Roseburia* in the gut of weaned piglets, Torres-Pitarch et al. [31] found that cecal *Roseburia faecis* was positively correlated with ADG in older pigs. In the current study, the increase in both the genus *Roseburia* and *Roseburia hominis* in the feces of piglets with the highest Mn supplementation serves as preliminary evidence that supplementary dietary manganese may foster the growth of the beneficial genus *Roseburia* in the gastrointestinal tract of piglets 35 d post-weaning, resulting in an increase in ADG.

The recent research has indicated that the genus *Turicibacter* can be beneficial to the health of the gastrointestinal tract; however, there is limited research as to how Mn affects this bacterium. *Turicibacter* was linked with increased intestinal butyrate in rats fed high-fat diets [41]. In swine production, *Turicibacter* was increased in pigs fed increased starch diets, which investigators concluded led to an increase in metabolic pathways that were beneficial to the host’s gastrointestinal health [42]. Additionally, the research has identified *Turicibacter* as an important bacterium for increasing body weight in pigs [43]. The present study’s results that the relative abundance of *Turicibacter* is maintained from d 0 to d 35 in the group with the highest Mn supplementation suggests the inclusion of dietary Mn can have positive impacts on the growth of this genus, ultimately increasing body weight while maintaining gastrointestinal health in weaned piglets.

*Acidaminococcus fermentans* was isolated by Fuller [44] from a porcine alimentary tract. *A. fermentans* thrives primarily via glutamate fermentation in the gastrointestinal tract of animals, including pigs [44,45]. Although the previous research has not investigated the impact of trace minerals on the gastrointestinal tract, it is well supported that dietary fiber inclusion rates impact the gut microbial populations [46,47]. Pigs fed a low fiber diet had increased abundances of *A. fermentans* [48]. They concluded that dietary fiber modulates the host microbial population toward more beneficial butyrate-producing bacteria instead of more pathogenic bacteria including *A. fermentans*, *Clostridium perfringens*, and *Clostridium rectum*. The present study’s findings suggest Mn inclusion may have similar impacts on *A. fermentans* as fiber inclusion, since the piglets receiving the highest level of Mn supplementation had the lowest levels of *A. fermentans* on d 35. This further supports the hypothesis that Mn positively impacts host gastrointestinal health by decreasing pathogenic bacteria.

*Lactobacillus ruminis* is a commensal species of bacteria found in the gastrointestinal tract of several species of animals, including monogastric animals such as pigs [49]. In pigs, *L. ruminis* is considered to be the primary lactic acid bacteria in the large intestine [49,50]. Various species of *Lactobacillus* are considered to be beneficial to the pig and are used as feed additives either as pure or mixed cultures [51,52]. However multiple studies have found *L. ruminis* to be associated with increased levels of either interleukin-6 (IL-6) and IL-8, leading to an increase in inflammation [53,54]. This study may provide preliminary evidence that Mn supplementation may reduce fecal relative abundance of *L. ruminis* in piglets 35 d post-weaning, potentially reducing gut inflammation.

*Massiliomicrobiota timonensis* is a bacteria that is Gram-negative, rod-shaped and found in chains [55]. Little is known about *M. timonensis*. It was first isolated from an 87-year-old patient hospitalized in 2015 for cognitive impairment experiencing weight loss complications [55]. To the best of our knowledge, this was the first time *M. timonensis* was isolated in the feces of pigs. Combining previous findings that the patient was experiencing weight loss at the time of isolation with the fact that in the current study, the piglets that had an increased relative abundance of *M. timonensis* had a lower ADG, an argument could be made that this bacterium might be associated with reduced nutrient absorption. This could be attributed to the host having a less “healthy” gut. Although additional research is required to determine the specific function *M. timonensis* plays in the gastrointestinal microbial consortium, the current data suggest that *M. timonensis* may possess pathogenic tendencies by being more abundant in less efficient piglets who also had increased relative abundances of other pathogenic bacteria.

## 5. Conclusions

The results from this research trial highlight the importance of Mn supplementation on the increasing growth of nursery piglets following weaning. There were no statistically significant effects observed in relation to selenium or the interaction of manganese with selenium. This was not unexpected, as adequate vitamin E was supplemented in all diets, which would have negated any negative effects observed in a lower selenium diet. Although dietary concentrations of manganese did not alter the fecal microbiota as a whole from a microbial diversity perspective, it did have an effect on specific microorganisms. Although the present nursery trial had a relatively small sample size, it highlights the importance for potential research into how Mn impacts the gut microbial populations. Specifically, our data suggest Mn may have some antimicrobial effects by reducing key bacteria that exhibit pathogenic behavior and create gut inflammation, consequently decreasing growth rates. Additionally, dietary Mn supplementation may also foster the growth of beneficial microorganisms that increase body weight gain while maintaining gastrointestinal health. The present research trial results provide preliminary indications that increasing dietary Mn in nursery piglets (12 or 24 mg/kg) increases ADG and gut health. Additionally, the reduction of potentially harmful pathogenic microorganisms is of great importance to the food animal industry from a production and food safety perspective, and Mn may be a solution to the reduction of these pathogens.

## Figures and Tables

**Table 1:** Composition and analysis of basal diets.

Ingredients, %	Phase I ^a^	Phase II ^a^	Phase III ^a^
Corn	22.80	40.20	57.34
Soybean meal 47.5%	15.00	21.00	28.14
Whey	15.00	10.00	7.00
Oats	10.00	5.00	
Hamlet protein ^1^	10.00	7.50	
Lactose	10.00	3.00	
Fish meal	5.00	3.00	3.00
Blood plasma	3.00	1.50	
Soybean Oil	5.24	4.38	0.46
L-Lysine	0.32	0.40	0.30
DL Methionine	0.20	0.22	0.14
L-Threonine	0.12	0.16	0.12
Dicalcium phosphate	1.48	1.74	1.34
Limestone	0.32	0.38	0.64
Salt	0.26	0.26	0.26
UGA vitamin premix ^2^	0.26	0.26	0.26
Trace mineral premix ^3,4,5^	1.00	1.00	1.00
Nutrient Composition ^5^			
ME ^6,7^, kcal/kg	3511	3500	3319
Crude protein, %	21.1	21.2	21.3
Lysine ^7^, %	1.76	1.66	1.32
Crude fat, %	7.1	6.9	3.3
Ash, %	6.3	6.2	5.6
Crude fiber, %	1.6	7.4	7.7
Phosphorus (total), %	0.74	0.82	0.74
P (avail) ^7^, %	0.55	0.45	0.37
Calcium, %	0.97	1.1	0.93

^a^ The nursery diets were formulated to meet NRC requirements (2012). Phase I was fed for 7 d, Phase II for 14 d, and Phase III for 14 d. ^1^ HP 300; Hamlet Protein Inc., Findlay, OH 45840. ^2^ Vitamin premix: supplied per kg of diet: vitamin A (4,134 IU); vitamin D (1,653 IU); vitamin E (66 IU); vitamin K (3.3 mg); riboflavin (8.27 mg); niacin (49.6 mg); vitamin B_12_ (0.033 mg); pantothenic acid (27.6 mg); ADM Alliance Nutrition, Quincy, IL 62305. ^3^ The trace minerals (except Se and Mn) were provided at 20 mg/kg copper (CuSO_4_); 100 mg/kg iron (FeSO_4_); 100 mg/kg zinc (ZnO); 0.1 mg/kg iodine (KIO_3_). Ground corn was used as a carrier for the premixes. Analyzed values (mg/kg diet): 20.7 mg Cu; 398.7 mg Fe; 141.8 mg Zn. Iodine concentrations were too low to distinguish in the diet. ^4^ Selenium was supplemented at two concentrations: 0.1 or 0.3 mg/kg (Na_2_SeO_3_); Mn was supplemented at 0, 12, or 24 mg/kg (MnSO_4_) for each concentration of Se, resulting in six unique premixes that had an inclusion rate of 1% in each complete diet. The analyzed Mn was 28.3, 39.9, and 51.7 mg/kg for 0, 12, and 24 mg/kg supplemented Mn diets, respectively. The Se concentrations were too low to distinguish in the complete diets. ^5^ Proximate and mineral analysis was carried out by the Feed, Water, and Soil Laboratory at the University of Georgia. ^6^ Metabolizable energy. ^7^ Calculated value.

**Table 2:** The impact of varying dietary concentrations of supplementary manganese and selenium on the growth performance parameters of nursery pigs (*n* = 5 pens/treatment).

Treatments ^1^ Se, mg/kg	0.1			0.3			*p*-Values ^2^			
Mn, mg/kg	0	12	24	0	12	24	SEM	Mn	Se	Mn × Se
Body weight, kg										
d 0	5.12	5.33	5.19	5.19	5.26	5.24	0.7			
d 35	18.81	20.23	19.60	19.02	20.77	6.64	0.7	0.216	0.455	0.914
Performance d 0–35										
ADG (g/pig/day)	393 ^b^	429 ^ab^	414 ^ab^	397 ^b^	445 ^a^	442 ^a^	13	0.007	0.140	0.661
ADFI (g/pig/day)	541	584	583	547	559	581	22	0.221	0.702	0.767
Gain:Feed	0.73	0.74	0.71	0.71	0.80	0.76	0.03	0.144	0.141	0.191

^a,b^ LS Means within a row that do not share the same letter are significantly different from one another (*p* < 0.05). ^1^ Dietary treatments were created to follow a 2 × 3 factorial design. Within each concentration of supplementary Se (0.1 and 0.3 mg/kg), there were three concentrations of supplementary Mn (0, 12, 24 mg/kg). ^2^ *p*-values presented are representative of the main effects of manganese (Mn), selenium (Se), and the interaction of Mn and Se (Mn × Se). There was a significant linear effect of Mn on overall ADG (*p* < 0.05) but on no additional parameters throughout.

**Table 3:** The impact of varying dietary concentrations of supplemental manganese and selenium on volatile fatty acid profile and microbial diversity indices (*n* = 5 pens/treatment).

Treatments ^1^ Se, mg/kg	0.1			0.3			SEM
Mn, mg/kg	0	12	24	0	12	24	
VFA Concentration							
Acetate (MP ^2^) ^3,4^							
d 0	76.06 ^a^	68.48 ^ab^	61.16 ^c^	70.64 ^ab^	74.00 ^ab^	72.61 ^ab^	2.4
d 35	55.14 ^cd^	55.71 ^cd^	54.19 ^d^	54.17 ^d^	55.65 ^cd^	57.79 ^cd^	2.4
Propionate (MP ^2^) ^3,4^							
d 0	14.91	18.51	16.79	18.29	17.00	16.6	1.4
d 35	28.54	29.00	29.67	29.95	29.47	28.16	1.4
Butyrate (MP ^2^) ^3,4^							
d 0	4.92	8.22	9.54	6.79	5.37	6.45	1.49
d 35	11.55	10.78	10.98	10.90	10.40	9.87	1.49
Valerate (MP ^2^) ^3,4^							
d 0	4.11	4.79	12.52	4.28	3.64	4.34	1.74
d 35	4.78	4.51	5.16	4.97	4.48	4.19	1.74
A:P ^3,4^							
d 0	5.40	3.75	3.87	4.19	4.40	4.87	0.46
d 35	1.92	1.89	1.81	1.78	1.86	2.04	0.46
Total VFAs ^3,4^							
d 0	38.98	49.70	48.59	39.62	39.00	37.18	12
d 35	153.15	128.58	137.23	147.53	115.45	122.43	12
Alpha Diversity							
OTUs ^3,4^							
d 0	638	618	595	683	596	638	56
d 35	665	644	628	529	691	725	
Shannon ^3,4^							
d 0	7.84	7.51	7.79	7.87	7.73	7.85	0.21
d 35	7.79	7.72	7.49	7.42	7.70	8.00	
Evenness ^3,4^							
d 0	0.84	0.81	0.86	0.84	0.85	0.84	0.02
d 35	0.84	0.83	0.81	0.83	0.82	0.84	

^a–d^ LS Means within a row that do not share a letter are significantly different from one another (*p* < 0.05). ^1^ Dietary treatments were created to follow a 2 × 3 factorial design. Within each concentration of supplementary selenium (0.10 and 0.30 mg/kg) there were three concentrations of supplementary manganese (0, 12, and 24 mg/kg). ^2^ Molar proportion. ^3^ *p*-values presented are representative of the main effects of manganese (Mn), selenium (Se), their interaction (Mn × Se), and the interaction of Mn with day (Mn × day), respectively, for each parameter are listed at subscript 4 in the footnotes. ^4^ Acetate: 0.214, 0.064, 0.004, 0.045; Propionate: 0.703, 0.639, 0.147, 0.803; Butyrate: 0.758, 0.187, 0.326, 0.310; Valerate: 0.094, 0.071, 0.326, 0.093; A:P: 0.499, 0.721, 0.083, 0.437; Total VFAs: 0.298, 0.144, 0.750, 0.090; OTUs: 0.903, 0.705, 0.359, 0.209; Shannon: 0.739, 0.561, 0.333, 0.544; Evenness: 0.570, 0.607, 0.712, 0.598.

## Data Availability

The nucleotide sequencing data were deposited in a public repository (www.mg-rast.org) under accession number mgm4906046.3. Deposited on 21 March 2021.

## Supplementary Materials

The following supporting information can be downloaded at: https://www.mdpi.com/article/10.3390/vetsci10110650/s1, Supplemental Figure S1: The effect of varying dietary on bacterial relative abundance on the genera *Streptococcus*, *Roseburia*, and *Turicibacter* in nursery pigs.
