# Supplementary material for: The Effects of Dietary Manganese and Selenium on Growth and the Fecal Microbiota of Nursery Piglets
Source: Vet Sci. 2023 Nov 10;10(11):650. doi: 10.3390/vetsci10110650 (PMC10675067; doi:10.3390/vetsci10110650)
Supplement: Supplementary file 1 [file vetsci-10-00650-s001.zip › vetsci-2659806-supplementary.pdf]

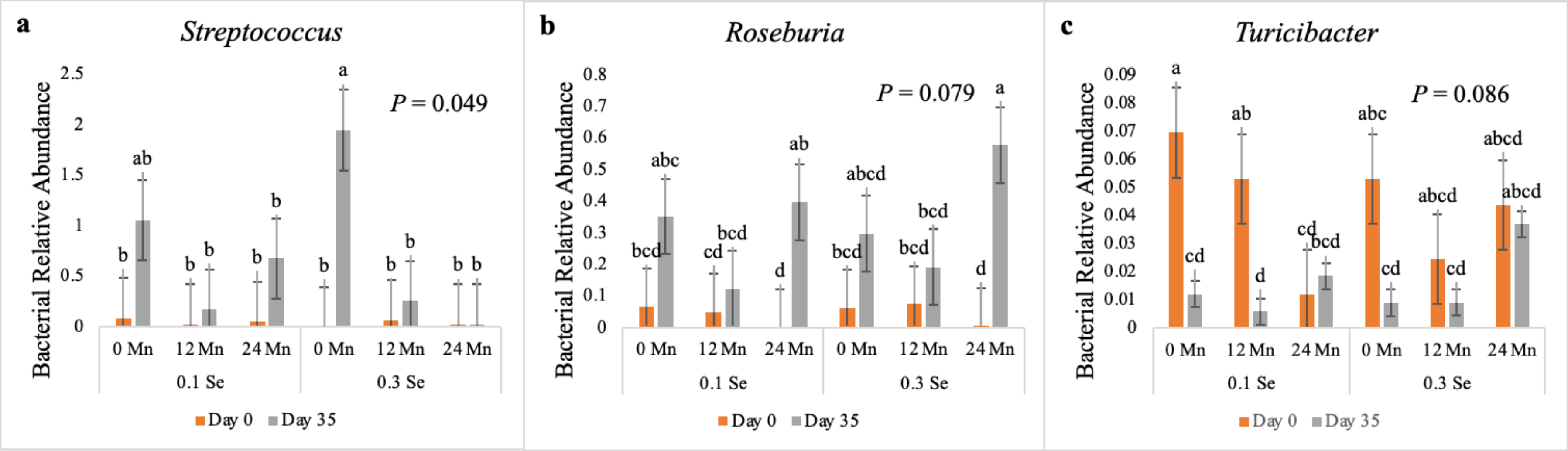

Supplemental Figure S1. The effect of varying dietary manganese and selenium on bacterial relative abundance for the genera *Streptococcus* (**a**), *Roseburia* (**b**), and *Turicibacter* (**c**) in nursery pigs (n = 30 pens). *P* values reported for MnXDay effect. [abcd] indicates significant differences ($P < 0.05$).
